# Predictive Brain Signals of Linguistic Development

**DOI:** 10.3389/fpsyg.2013.00025

**Published:** 2013-02-08

**Authors:** Valesca Kooijman, Caroline Junge, Elizabeth K. Johnson, Peter Hagoort, Anne Cutler

**Affiliations:** ^1^Food and Biobased Research, Wageningen University and Research CentreWageningen, Netherlands; ^2^Department of Psychology, University of AmsterdamAmsterdam, Netherlands; ^3^Department of Psychology, University of Toronto MississaugaMississauga, ON, Canada; ^4^Max Planck Institute for PsycholinguisticsNijmegen, Netherlands; ^5^Donders Institute for Brain, Cognition and Behaviour, Radboud University NijmegenNijmegen, Netherlands; ^6^MARCS Institute, University of Western SydneyPenrith, NSW, Australia

**Keywords:** infant speech perception, speech segmentation, language skill development, vocabulary size, brain development, brain polarity, ERPs

## Abstract

The ability to extract word forms from continuous speech is a prerequisite for constructing a vocabulary and emerges in the first year of life. Electrophysiological (ERP) studies of speech segmentation by 9- to 12-month-old listeners in several languages have found a left-localized negativity linked to word onset as a marker of word detection. We report an ERP study showing significant evidence of speech segmentation in Dutch-learning 7-month-olds. In contrast to the left-localized negative effect reported with older infants, the observed overall mean effect had a positive polarity. Inspection of individual results revealed two participant sub-groups: a majority showing a positive-going response, and a minority showing the left negativity observed in older age groups. We retested participants at age three, on vocabulary comprehension and word and sentence production. On every test, children who at 7 months had shown the negativity associated with segmentation of words from speech outperformed those who had produced positive-going brain responses to the same input. The earlier that infants show the left-localized brain responses typically indicating detection of words in speech, the better their early childhood language skills.

## Introduction

Spoken language is one of the dimensions of the infant’s environment for which perceptual information is available, processed, and stored even before birth (DeCasper et al., 1994). Accordingly, the first year of an infant’s life sees steady continuous growth in the skills required to turn a speech signal into a comprehended message (Saffran et al., 2006). Although the first spoken words may be produced only at the end of that year, the perceptual skills that make such production possible develop steadily from birth onward.

This development is not simply a passive result of maturation. The infant’s task is to acquire the environmental language(s), and thus to attend to meaningful perceptual variation where it is required to differentiate relevant contrasts (and accordingly to ignore variation that is perceptually detectable, but irrelevant to this particular language). Differences between languages and acoustically salient differences within a language induce differences in the speed and the order with which this phonological task is achieved (Narayan et al., 2010).

One of the most important skills an infant must acquire is the ability to segment speech, i.e., to recognize a word form even though it is embedded in a speech context that may be completely novel. Since speech input to infants consists mainly of multi-word utterances (Van de Weijer, 1999), segmentation is a vital prerequisite of initial vocabulary construction, and infants indeed display segmentation skills before they command a workable vocabulary. This was demonstrated by Jusczyk and Aslin (1995), using a two-phase Headturn Preference Procedure (HPP). Infants were first familiarized with words in isolation, then tested with short texts which did or did not contain the familiarized words. Infants listened longer to the texts containing the target words than to the control texts, showing that they could indeed distinguish between the two – in other words, that they had detected the target words although they were embedded in continuous speech.

Phonological differences between languages also affect the relative appearance of segmentation abilities in the two-phase HPP. English and Dutch are very closely related languages, but evidence of segmentation skills has been seen earlier in English than in Dutch HPP studies, and this difference is ascribed to the relative salience of the cues involved. Across languages, cues to word segmentation can be derived from characteristic rhythmic patterns, and both adults and infants exploit this correspondence in parsing speech (Cutler, 1994). In English, rhythm is stress-based and essentially reduces to the distinction between strong syllables with full vowels and weak syllables with reduced vowels (Fear et al., 1995). This makes for an easy and salient distinction, and English-acquiring infants show segmentation skills in HPP for monosyllabic or bisyllabic initially stressed nouns at least by 7.5 months (Jusczyk and Aslin, 1995; Jusczyk et al., 1999). In Dutch, rhythm is also stress-based, but the strong-weak distinction is less salient than in English, since vowels in unstressed syllables are less frequently reduced (Van der Hulst, 1984). Dutch babies segment speech successfully at 9 months, but fail to show segmentation at 7.5 months (Kuijpers et al., 1998; note that this study was a direct Dutch replication of Jusczyk et al., 1999).

There is also variation across individuals. This variation is related to later language development, as Newman et al. (2006) discovered. They collected vocabulary scores at 2 years for children who had taken part in various HPP segmentation experiments in their first year, and selected from the extensive group the 15% with the largest vocabularies (on average 646 words) and the 15% with the smallest vocabularies (on average 73 words). Members of the former group were significantly more likely as infants to have shown a segmentation effect, in line with the group pattern, than their age mates who now had lower vocabulary scores. Results from experiments that did not involve segmentation, for instance discriminating between languages, were unrelated to later vocabulary size. Newman et al.’s finding is important, as it was the first to underscore the close relationship between being able to segment words from speech and being able to store words in a vocabulary.

Although Newman et al. (2006) drew their conclusion from a comparison of the two outer ends of a large vocabulary size distribution, Singh et al. (2012) showed that the same relationship held across a group of 40 individuals. Singh et al. tested infants at 7.5 months with a simple segmentation task (as used by Jusczyk and Aslin, 1995) and a more complex segmentation task in which the familiarization stimuli could differ from the test stimuli in pitch, then tracked their vocabulary growth to age two. At an individual level, recognition scores (the difference in listening time across trials with familiarized versus unfamiliarized input) on each task correlated significantly with vocabulary size at 24 months, with the more complex task showing stronger correlation. The better the 7.5-month-olds’ segmentation skills, the more words they knew at age two.

Recent findings further suggest that a link between early speech segmentation ability and later vocabulary size also holds for preterms: although as a group they do not demonstrate similar evidence of segmentation skill compared to full term 8-month-olds (matched for gestation), those who show similar behavioral responses have higher productive vocabulary at 12 and at 18 months (Bosch, 2011).

In the HPP, the duration of an infant’s behavioral response (a headturn to keep listening to an audio input) provides evidence that familiarized words have been detected and thus that segmentation has happened. This is a reliable indicator of segmentation, but it is not a direct view of the segmentation in process. It became possible to track segmentation as it happens, however, once a version of the two-phase segmentation experiment was developed that was suitable for use with measurement of event-related potentials (ERPs). In an ERP study, brain responses time-locked to onset of a familiarized word can be compared with responses to a control word that was not heard before. Kooijman et al. (2005) devised such a method; they tested Dutch 10-month-olds, using low-frequency Dutch bisyllabic words of the kind that Kuijpers et al. (1998) had used in their Dutch HPP study. Familiarization with 10 occurrences of the same word (e.g., *monnik* “monk”; see Table 1) in isolation produced a response that became steadily more negative. After familiarization with a word, the infants heard eight sentences, four containing the familiarized word and four a matched control word. Infant brain responses keyed to the onset of familiarized target words were significantly negative in amplitude relative to the responses to the unfamiliarized control words; that is, this difference in the infant brain responses as the spoken sentences were being heard was here the measure showing that a familiar word had been detected.

**Table 1:** **An example of an experimental trial in the ERP study, with English glosses**.

Familiarization	Ten tokens of either *monnik* or *sultan*
Test	*De* *monnik wiedt zijn tuintje dagelijks*
	“The monk weeds his garden every day”
	*De strenge* *sultan regeert met straffe hand*
	“The strict sultan rules with an iron hand”
	*De* *sultan bestuurt het kleine landje*
	“The sultan administers the little country”
	*Pieter ziet de vriendelijke* *monnik in het hofje*
	“Peter sees the friendly monk in the almshouse”
	*Volgend jaar komt de jonge* *sultan naar Nederland*
	“Next year the young sultan is coming to The Netherlands”
	*Omar geeft de vriendelijke* *sultan nog een sigaar*
	“Omar gives the friendly sultan another cigar”
	*Elke week plukt de jonge* *monnik verse appels*
	“Every week the young monk picks fresh apples”
	*De strenge* *monnik draagt een zware habijt*
	“The strict monk wears a heavy habit”

*The experimental words are underlined in the sentences; the word that was heard in familiarization was deemed the familiar word, its pair was then the unfamiliar control*.

Subsequent studies confirmed that the stress-based segmentation underlying the HPP results also drove the negative-going ERP segmentation response (Kooijman et al., 2009), and showed significant evidence of segmentation by some 10-month-olds even without prior familiarization: presented first with a sentence such as *De strenge monnik draagt een zware habijt* “The strict monk wears a heavy habit,” these infants then produced the negative-going recognition response to *monnik* presented later in isolation (in comparison to a control word that had not been part of the preceding sentence; Junge et al., 2012).

Further ERP research on speech segmentation also showed more negative-going brain responses for familiarized words relative to unfamiliar control words in (older) infants acquiring other languages. A negative familiarity effect was observed in 12-month-olds acquiring European French (Goyet et al., 2010; this study used familiarization with isolated words and a test phase of target words in passages, as in Kooijman et al., 2005). The same effect was observed in German 12-month-olds, in a study using familiarization with words within passages and test with isolated words (Männel and Friederici, 2010).

Just as the HPP segmentation response is related to later vocabulary development, so is the ERP segmentation response. Of the 28 infants tested by Junge et al. (2012), 18 showed the ability to achieve segmentation without prior familiarization, while 10 did not. In line with Newman et al.’s (2006) and Singh et al.’s (2012) evidence from HPP studies, a *post hoc* analysis of Junge et al.’s (2012) ERP data showed a relationship between vocabulary size at 12 months and the presence of this segmentation ability at 10 months. A median split was applied to vocabulary measures collected at 12 months via parental questionnaires, yielding a group with larger receptive vocabularies at that age (mean 146 items; range 71–264) and a group with smaller vocabularies (mean 40, range 0–68). In the sentence familiarization task, the former group showed a significant negative recognition response; the latter group did not. In a condition where one isolated word was presented both in familiarization and test, so that word segmentation abilities were not required, each group showed evidence of word recognition. Thus the online ERP measure offers insight into individual differences in success at early word recognition tasks requiring speech segmentation, and how these differences relate to language learning in general.

The ERP studies described so far have shown segmentation at 10–12 months, but HPP studies have shown segmentation to occur earlier, at 7.5 or 8 months (Jusczyk and Aslin, 1995; Polka and Sundara, 2012). The online ERP measure, requiring no behavioral response from infants, may hence allow a more direct reflection of Dutch infants’ segmentation capacities, at an earlier age than so far demonstrated with the HPP. However, the literature on infant ERPs shows that responses are quite likely to vary as a function of age. For example, early responses can manifest with different polarity from responses later in life. Kudo et al. (2011) report a positive-going response indicating segmentation of a sequence of tones by neonates, where the same sequences had produced detection negativities in adults (Abla et al., 2008). Männel and Friederici (2010) found that 6-month-old German-learners showed a positivity in a familiarization condition that required segmentation ability, while in 12-month-olds the same condition elicited a clear negative response. Likewise, in an ERP study of phonetic discrimination responses Garcia-Sierra et al. (2011) found that infants acquiring both English and Spanish tended at 6–9 months to show a positive-going response to phonetically deviant stimuli, whereas at 10–12 months the same stimuli elicited negative-going responses. Indeed, in the original Kooijman et al. (2005) ERP study, not all participants showed the negative-going recognition response that constituted the average result. A minority showed, instead, a positive-going response to the target words at test (Junge, 2011).

Polarity differences across age groups in infancy can simply reflect differing relations of a constantly placed reference electrode to a test electrode on a very small versus a larger skull. They can also arise from maturation effects; ERP maturation from birth to the first birthday shows an overall pattern in which the generators responsible for positive amplitudes mature earlier (in the first 6 months) than those responsible for negative amplitudes (from 6 months on; Kushnerenko et al., 2002). In both cases, it is unlikely that observed polarity differences in ERPs to speech signals relate systematically to underlying cognitive processes. In contrast, a third possibility could be that polarity differences reflect differences in relative task demands or in auditory processing (Rivera-Gaxiola et al., 2005b). We will return to this issue in the discussion.

In this paper we report an ERP study of word segmentation from continuous speech by Dutch infants at 7 months. This is a particularly interesting age given that American English learners can segment speech in HPP studies at 7.5 months (Jusczyk and Aslin, 1995; Jusczyk et al., 1999) and their abilities at that age are related to their later vocabulary size (Singh et al., 2012), while Dutch learners at that same age do not demonstrate segmentation ability in HPP (Kuijpers et al., 1998). The ERP paradigm, though, provides a more sensitive view of learners’ early responses to language input. We report detailed analysis of ERP patterns associated with segmentation in our study with 7-month-olds, and assessment of the subsequent language abilities of the same participants at 3 years. From this we conclude that early ERP patterns indexing speech segmentation ability directly predict later patterns of language skills.

## ERPs at 7 Months

### Participants

Twenty-eight 7-month-old infants from Dutch monolingual families participated (mean age = 7.05 months; age range = 6.11 −7.19 months; 13 female). Twenty-two additional infants were tested, but excluded from data analyses because of fussiness or sleepiness. All infants were reported to have normal development and hearing, and no major problems during pregnancy or birth. All infants were full term, bar one who had been 3.6 weeks premature. There were no neurological or language problems in the immediate families. The parents signed a consent form and received 20 euro for participation.

### Stimuli and design

We used the same stimuli and design as in Kooijman et al. (2005). Forty low-frequency bisyllabic initially stressed nouns were selected from the CELEX Dutch lexical database (Baayen et al., 1993); examples are *monnik* “monk,” *sultan* “sultan.” A set of four sentences was constructed for each noun. The nouns were arranged in pairs, with noun position in the sentences, and words preceding the noun, matched across pairs; Table 1 shows an example noun pair with corresponding sentences. The stimuli (all the sentences, and 10 isolated tokens of each noun) were recorded in a sound-attenuating booth by a female speaker of Dutch in a lively child-directed manner, and sampled to disk at 16 kHz mono. The mean duration of the nouns was 710 ms for the isolated words (range: 373–1269 ms) and 721 ms for the target words in the sentences (range 224–1046 ms). The sentences had a mean duration of 4082 ms (range: 2697–5839 ms).

The experiment contained 20 experimental familiarization + test trials (for an example see Table 1), each with 10 tokens of a target noun (familiarization), followed by eight randomized sentences (test). Four of the test sentences contained the word just familiarized (familiarized target words); four contained the unfamiliar noun paired with it (unfamiliar control words). There were four presentation lists, counterbalancing familiarization set (half of the target words were used for familiarization in Lists A and B, the other half in Lists C and D) and Order of presentation (Lists B and D were as A and C, but with the trials ordered inversely). Each list was heard by seven infants.

### Procedure

Infants were seated in a child seat in a sound-attenuating test booth and listened to the stimuli via three loudspeakers situated to the front. Also in front of the infants, a computer screen showed a moving screensaver, not synchronized with the stimuli, and the infants could additionally play with a small silent toy. A parent sat next to each child and listened to a masking CD through closed-ear headphones. Breaks were taken when necessary. Familiarization and test blocks were presented until an infant became too distracted to continue. The experiment lasted on average 32 min; mean block length was 1.6 min, with 2.5 s silence between isolated words and 4.2 s silence between sentences. Subjects heard at least eight blocks (mean: 13, range: 8–20).

### EEG recordings

Electroencephalogram (EEG) measurement was via infant-size Brain-Caps with 27 Ag/AgCl sintered ring electrodes. Twenty-one electrodes were placed according to the American Electroencephalographic Society 10% standard system (midline: Fz, FCz, Cz, Pz, Oz; frontal: F7, F8, F3, F4; fronto-temporal: FT7, FT8; fronto-central: FC3, FC4; central: C3, C4: centro-parietal: CP3, CP4; parietal: P3, P4; and occipital: PO7, PO8). Six electrodes were placed bilaterally on non-standard positions: a temporal pair (LT and RT) at 33% of the interaural distance lateral to Cz, a temporo-parietal pair (LTP and RTP) at 30% of the interaural distance lateral to Cz and 13% of the inion-nasion distance posterior to Cz, and a parietal pair (LP and RP) midway between LTP/RTP and PO7/PO8.

The left mastoid served as online reference for all electrodes. EEG electrodes were referenced to the left mastoid online and re-referenced offline to linked mastoids. Vertical eye movements and blinks were monitored via a supra- to sub-orbital bipolar montage, and horizontal eye movements via a right-to-left canthal bipolar montage. Two occipital electrodes (PO7, PO8) and the midline electrodes Fz, FCz, Cz, Pz, Oz were excluded from analysis either due to excessive artifact (mainly the parietal and occipital electrodes, because the infant’s back of the head rested against the child seat) or due to poor cap fit (for some of our subjects we could not get good recordings from FCz and Cz, because all electrodes were bundled together above Cz, creating too much space between the fronto-central electrodes and the skull). Impedances at the remaining electrodes were around 10 kΩ. A BrainAmp DC EEG amplifier recorded EEG and EOG data using a band pass of 0.1–30 Hz and a sample rate of 200 Hz. Excess slow wave activity can often obscure ERP effects in young infants (Weber et al., 2004); to remove it, we filtered the EEG signal offline to 1–30 Hz before further analysis.

Offline, individual trials were aligned 200 ms before acoustic onset of the target words, and screened for artifact from −200 to 800 ms. We rejected trials when amplitude on any electrode channel exceeded ±150 μV or when clear correlations with the eye channels were observed. This resulted in rejection rates of 55.6 and 62.5% of the trials time-locked to the isolated words or to the target words in the sentences, respectively; these are similar rejection rates as in Kooijman et al. (2005). Infants contributed on average 11.4 (SD 3.0) artifact-free trials for the familiarization phase and 19.6 (SD 7.0) for the test phase.

### Statistical analyses

We examined the role of word familiarity for the familiarization phase (comparing ERPs for the first two isolated tokens (“unfamiliar”) versus the last two isolated tokens of the target noun (“familiarized”) and for the test phase (comparing ERPs to the four familiarized target versus the four unfamiliar control words within sentence context). For each condition for each subject, average waveforms were calculated in the −200 to 800 ms window. For illustration purposes, we averaged for each condition the subject average waveforms into grand average waveforms. The number of trials used in each grand average waveform was respectively 332 and 309 for the unfamiliar and familiarized isolated words, and 549 and 548 for the unfamiliar control and familiarized target words in the sentences. Time windows for statistical analyses were chosen based on visual inspection of the data.

Repeated measures analyses of variance were performed for the chosen time windows with Familiarity (two: Familiar; Unfamiliar), Quadrant (four: Left Frontal; Right Frontal; Left Posterior; Right Posterior), and Electrode (five per quadrant; Left Frontal: F7, F3, FT7, FC3, C3; Right Frontal: F8, F4, FT8, FC4, C4; Left Posterior: LT, LTP, CP3, LP, P3; Right Posterior: RT, RTP, CP4, RP, P4) as within-subject variables. The Huynh–Feldt epsilon correction was used for all tests. The original degrees of freedom as well as the adjusted *p*-values are reported. The onsets of the effects were tested by performing *t*-tests on the difference waveforms in bins of 50 ms with a 40 ms overlap (i.e., 0–50, 10–60 etc), with significance from zero (*p* < 0.05) on five consecutive bins taken as evidence for onset.

### Results: Isolated words

**Figure 1:**
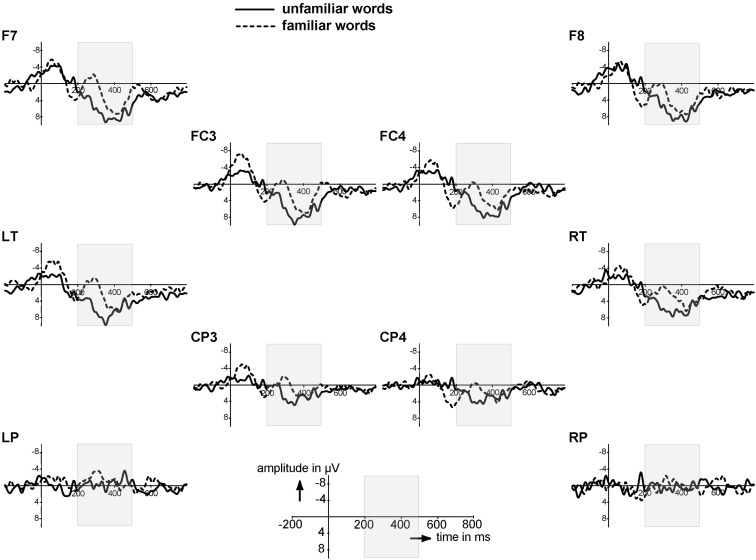
**Event-Related Brain Potentials to the unfamiliar (word position 1 & 2) and familiar (word position 9 &10) isolated words on a subset of electrodes; negativity is plotted upwards**. Electrodes are laid out as they are on the scalp. The gray area indicates the time window of 200–500 ms.

The isolated words allow assessment of sensitivity to repetition. We averaged the EEG to token 1 and 2 of the familiarization phase, representing the ERP response to the most unfamiliar isolated words, and the EEG to token 9 and 10, representing the ERP response to the most familiar of the isolated words because by then eight tokens of the same word had already been heard. A difference between these two averages signals an infant’s recognition of the repetition. The ERPs to these unfamiliar versus familiarized isolated tokens indeed seem to differ in two time windows, as Figure 1 shows. First, there is one early peak from 40 to 20 ms that is more negative to the familiarized than to the unfamiliar tokens over a subset of electrodes (FC3, FC4, LT, CP3). Second, familiarized isolated words elicited again a more negative ERP than unfamiliar isolated words in the 200–500 ms time window, mainly over frontal electrodes. This is in the same time window, and with similar distribution and polarity, as the familiarity effect for isolated words reported for the older age group (Kooijman et al., 2005). We analyzed the mean amplitudes in these time windows.

The first time window, the N1, did not show significant differences (*F*_1,27_ = 2.43, *p* = 0.13; no significant interactions with Familiarity). We then examined the same time window (200–500 ms) as in Kooijman et al. (2005) for the familiarization phase. There was an effect of Familiarity that narrowly missed significance (*F*_1,27_ = 3.39, *p* = 0.077), and a significant interaction of Familiarity with Quadrant (*F*_3,81_ = 2.74, *p* = 0.05). Analyses per quadrant revealed a main effect of Familiarity over the left frontal quadrant only (*F*_1,27_ = 5.94, *p* = 0.02); the right frontal and the posterior quadrants showed no significant effects (*p* > 0.10). Thus, the broad negative ERP effect to the familiar isolated words is strongest over the left frontal area. Onset analyses (see Statistical Analyses) revealed an onset starting at 220 ms for the left frontal electrodes F7 and FT7.

These ERP results thus show a brain response to the repetition of tokens of the same word starting at 220 ms. This familiarity response is similar in polarity and in distribution to that found by Kooijman et al. (2005), but starts 60 ms later; 10-month-olds in that study showed a Familiarity response starting at 160 ms. Like the 10-month-olds, however, the present 7-month-old listeners can recognize repetition of the same form in isolation, a prerequisite for being able to detect repetition of the same form in a speech context.

### Results: Sentences

**Figure 2:**
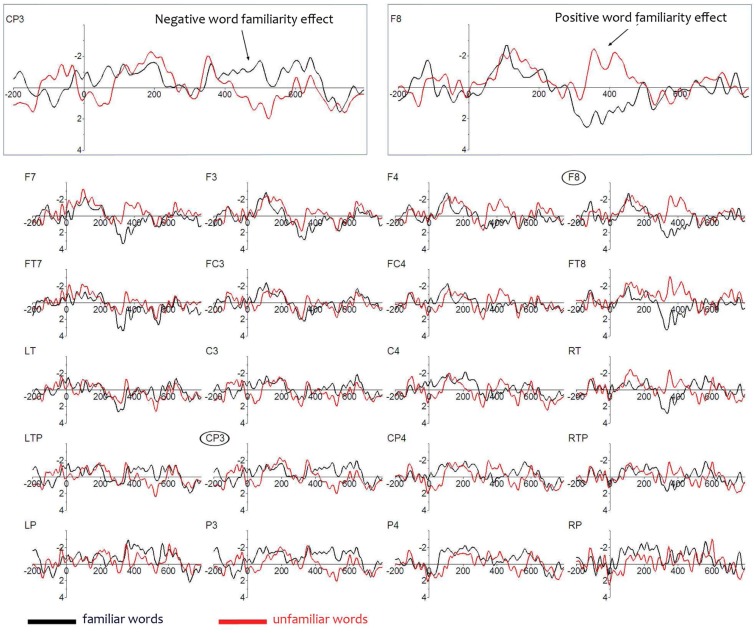
**Event-Related Brain Potentials on lateral electrodes to the familiarized target and unfamiliar control words in the sentences; negativity is plotted upwards**. Electrodes are laid out as they are on the scalp. Enlarged are a left centro-parietal electrode (CP3) illustrating the later negative familiarity effect, and a right frontal electrode (F8), illustrating the earlier positive familiarity effect.

Figure 2 shows that the ERPs to the familiarized target and unfamiliar control words in the sentences deviate from each other in two ways. First, familiarized target words elicit a more positive ERP than unfamiliar control words over the frontal areas from 350 to 450 ms, and second, they elicit a more negative ERP than unfamiliar control words over the left posterior area starting at about 430–530 ms. We performed statistical analyses over the mean amplitudes in these time windows.

A significant interaction of Familiarity × Quadrant (*F*_3,81_ = 4.05, *p* = 0.018) was observed for the 350–450 ms window, but there was no main effect of Familiarity (*F*_1,27_ < 1). Analyses per quadrant showed a narrowly missed significant effect of Familiarity over the right frontal quadrant (*F*_1,27_ = 3.70, *p* = 0.065), suggesting a more restricted location of the effect within this quadrant. Further analyses over a subset of four electrodes (F4, F8, FC4, and FT8) in that quadrant indeed revealed a significant main effect of Condition (*F*_1,27_ = 4.28, *p* = 0.048). There were no significant effects in equivalent analyses for the remaining three quadrants. Seventeen participants showed a positive effect on right frontal electrodes. Thus, the early effect of Familiarity is strongest over the right frontal brain area and has a positive polarity. Onset tests revealed a significant effect (*p* < 0.05) at 300 ms for electrode FT8.

In the later time window (430–530) statistical analyses show no significant main effect of Familiarity (F_1,27_ < 1) and no interaction between Quadrant and Familiarity (*F*_3,81_ = 2.31, *p* = 0.10). Visual inspection of the grand average waveforms reveals that in this window the effect is restricted to electrodes over the left hemisphere at the posterior sites LTP, CP3, and P3. An analysis over only these three left posterior electrodes revealed a significant effect of Familiarity (*F*_1,27_ = 4.24, *p* = 0.049; 14 participants showed this effect). In sum, we observe in the test phase two rather localized effects: a positive right frontal effect and a negative left posterior effect.

These two effects could be equally present in all children, such that the same children who show a positive right frontal effect are also the ones who show a negative left posterior effect. Another possibility could be that there are two subpopulations: some infants show a positive frontal effect yet others a negative left-going effect.

To examine whether the two familiarity effects in the test phase come from distinct or from the same populations, we calculated the correlation between these two effects (i.e., the average difference in amplitude from the four right frontal electrodes in the early time window with the average difference in amplitude from the three left posterior electrodes in the later time window). A (significant) positive correlation would be evidence of two subpopulations, whereas a negative correlation would indicate that the positive and the negative familiarity effects would be nearly simultaneously present within the same population. Indeed, there was a significant positive relationship [*r*(28) = + 0.41, *p* = 0.03], suggesting that the two effects are not driven by the same participants: those with an early positive familiarity effect continue to have a positive familiarity effect, and those with a later negative familiarity effect did not have an earlier positive effect. This could also explain why we do not find a significant effect on left fronto-temporal electrodes, which was the site at which the familiarity effects for 10-month-olds were observed (Kooijman et al., 2005; Junge et al., 2012): the different polarities of the familiarity effect on left frontal electrodes for each sub-group would cancel each other out in a grand average.

**Figure 3:**
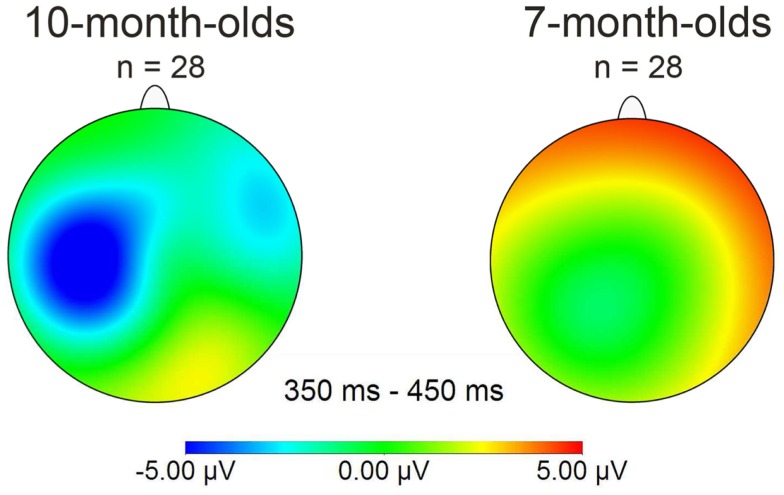
**Mean distribution plots for the ERP effect of familiarity (familiarized target – unfamiliar control words) in the 350–450 ms time window for overall group performance of 10-month-olds (left) and 7-month-olds (right)**.

Together, this suggests that our Dutch 7-month-old participants fall into two separate sub-groups, each showing evidence of being able to detect words previously heard in isolation when they re-occur in continuous speech. Note that word segmentation skill is here demonstrated in Dutch infants at an age at which behavioral evidence of segmentation is not available (Kuijpers et al., 1998)^1^. A majority of 7-month-olds demonstrated being able to segment words by showing a positive familiarity effect on right frontal electrodes. However, as Figure 3 shows, this effect differs in polarity (positive instead of negative) as well as in distribution (on right frontal instead of on left electrodes), compared to other studies reporting word familiarity effects indexing word segmentation skill in 10-month-olds (Kooijman et al., 2005; Junge et al., 2012).

Nevertheless, the two age groups both show a negative familiarity effect for the familiarization phase, during which the infants were not required to segment words from speech. Moreover, one sub-group among the present 7-month-olds also showed a negative familiarity effect when speech segmentation skill was required. This makes it unlikely that brain maturation underlies this polarity difference observed between the 7- and 10-month-olds, which was only present for the continuous speech condition. We will return to this issue in the general discussion. In the following section we first examine whether the polarity differences in our participant population are related to later language development.

## Language Skills at 3 Years

### Participants

Of the 28 participants in the ERP experiment, two could no longer be reached and the parents of a further three declined to participate; 23 children (82%) thus returned for further testing. These children (all right-handed; 11 girls) were now on average 36.3 months of age (range 28.4–46.6 months).

We first examined whether this subset of 23 participants continued to show an overall negative familiarity effect for isolated words in the 200–500 ms time window (familiarization phase), yet an overall positive familiarity effect for the words within speech in the 350–450 ms time window (test phase). Analyses revealed again a significant negative familiarity effect for the familiarization phase (*F*_1, 22_ = 5.61, *p* = 0.027), which was most pronounced over frontal electrodes (mean difference over frontal electrodes −3.71 μV, SD = 6.2). For the test phase, which required infants to segment words from speech, there was again no main effect of Familiarity (*F*_1,22_ < 1), but the interaction between Familiarity and Quadrant was significant (*F*_3,66_ = 5.17, *p* < 0.01). The familiarity effect is significant over the whole right frontal quadrant (*F*_1,22_ = 4.36, *p* < 0.05) and has a positive polarity (mean + 2.49 μV, SD = 5.7). Hence, even with a smaller sample we see a negative familiarity effect for the familiarization phase yet a positive one for the test phase. The subset of 23 children is thus representative of the full sample.

We then looked for polarity differences in their 7-month-old ERP results concerning the speech segmentation condition. We focused on results from this phase, because it is here that we observe polarity differences, not only between seven- versus 10-month-olds, but also within the 7-month-olds. Note moreover that Junge et al. (2012) only observed links between infant ERP measures of word recognition and later language development when infants had to first segment words from speech, not when they heard them first in isolation. In particular we inspected the polarity of each participant’s familiarity effect on left frontal electrodes, because it was on those electrodes that the familiarity effect was clearly present in 10-month-olds (Kooijman et al., 2005) and even turned out to be predictive of later vocabulary development in another sample of 10-month-olds (Junge et al., 2012). Moreover, as speculated in the previous section, a possible reason why we do not find any significant effect on left frontal electrodes for the 7-month-old overall analysis is that it is here that the two sub-groups overlap with their familiarity response (with reversed polarities), thereby canceling each other out. On this basis we identified two groups: nine “Negative responders” (three girls), with a negative-going ERP response resembling that found on average in both 10-month-old studies, and 14 “Positive responders,” whose response was positive-going as in the grand average of the ERP study. When we re-examined the time window 350–450 ms for the 23 subjects in the test phase, with Group as between-subjects variable, we observed, besides the significant interaction of Familiarity × Quadrant (*F*_3,63_ = 5.53, *p* = 0.003), two interactions with the factor Group: a significant Familiarity × Group (*F*_1,21_ = 24.3, *p* < 0.001), and a near-significant three-way Familiarity × Quadrant × Group (*F*_3,63_ = 2.67, *p* = 0.06). This shows that the two groups not only differ in polarity of the familiarity response, but also in the distribution of the effect. For the negative responders the familiarity effect had a negative polarity and was only significant in the left frontal and left posterior quadrants (*F*_1,8_ = 13.0, *p* < 0.01; *F*_1,8_ = 13.4, *p* < 0.01), whereas for the positive responders the familiarity effect had a positive polarity and was significant at *p* < 0.03 in all quadrants except the left posterior quadrant.

**Figure 4:**
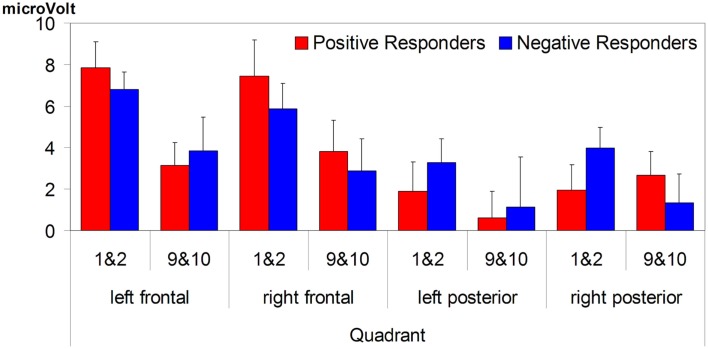
**Both groups show a similar decrease in positive amplitude for familiarized words in isolation (presented 9 & 10 times), compared to the first two times**. For both groups, the decrease was most pronounced over frontal quadrants of the brain.

We examined all other data available on the two groups. Two Positive responders (included in further analyses) reported having had speech therapy, and no Negative responders; but on no measure was there any significant difference between the two groups as a whole. Age did not differ (ERP experiment: positive responders mean age 217 days, Negative responders 218 days: *t*_21_ = −0,213, *p* = 0.83; follow-up testing: 37.6 and 34.4 months, respectively: *t*_21_ = 1,307, *p* = 0.21). Number of trials per condition in the ERP study did not differ: on average 21 trials per condition per Positive responder, and 20 trials per Negative responder (*t*_21_ = 0.55, *p* = 0.59; *t*_21_ = 0.10, *p* = 0.92 across familiar and unfamiliar words, respectively). Repetition effects in familiarization likewise did not differ: there were no significant interactions between Familiarity × Group for the first two versus the last two isolated word tokens in the familiarization phase (*F*_1,21_ < 1). Indeed, there were no polarity differences to be seen in the sub-groups’ responses at this stage of the ERP experiment. Figure 4 plots the response in μV for each sub-group to the first and the last pair of familiarization tokens, averaged for the 200–500 ms for each brain quadrant; it can be seen that there is a decrease in positivity (that is, a negative-going change) across familiarization that is virtually identical in average size for the two groups, and is further found for each group in each quadrant with only one exception (an insignificant shift in the opposite direction for Positive responders in the right posterior quadrant). This strongly suggests that our two sub-groups differ only in the abilities that are specifically needed for the ERP test phase but are not needed in familiarization.

**Figure 5:**
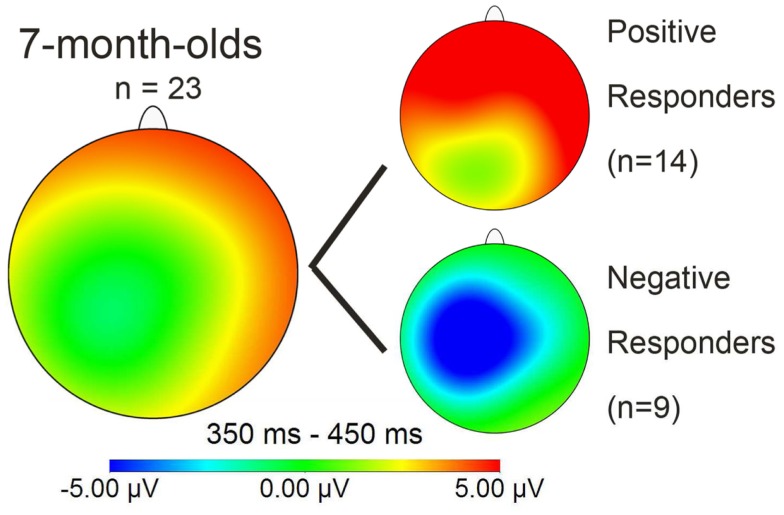
**Mean distribution plot for the ERP effect of familiarity (familiarized target – unfamiliar control words) in the 350–450 ms time window for those 7-month-olds who returned at 3 years for language testing; the two smaller plots divide the 7-month-olds into the sub-groups “Positive responders” and “Negative responders**.”

Similarity in latency across the groups was also evident in onset analyses for the test phase: in Positive responders, the familiarity effect had an onset at 100 ms for right electrodes FT8 and RT, in Negative responders at 110 ms for left electrodes FT7 and LT. In short, the polarity and the distribution of the ERP response pattern for words presented in continuous speech were the sole significant differences that we could find between the two sub-groups. The mean distribution plot for each group is displayed in Figure 5; comparison with Figure 3 makes clear that the Negative responder group deviates from the 28-participant seven-month average, and in fact closely resembles the pattern of negativity found with 10-month-old participants by Kooijman et al., 2005; see Figure 3 above.

### Language skill tests

We administered two norm-referenced language tests to all children: the *Reynell Test voor Taalbegrip* “test of language comprehension” (Van Eldik et al., 1995), and the Schlichting et al. (1995) *Test voor Taalproductie* “test of language production.” Together, the tests are a slightly modified Dutch translation of the Reynell (1985) Developmental Language Scales. They are the established scales used in the Netherlands for assessing language development problems, and are normed over 1,000 typically developing children. The test results for each child are converted into language quotients (LQs), with a mean of 100 and a SD of 15 points, that depend on the child’s age in months. An LQ below 85 is considered to indicate risk of language impairment. Both tests are graded in difficulty, allowing older children to start at a more advanced level, and both are suitable for children from 2 to 6 years.

The children were individually tested by the second author, unaware of their ERP profiles. In the first session they undertook the comprehension scale, in which they were asked to act out or point to requested objects. In the second session, scheduled on average 8 days (range 1–21 days) after the first session, they participated in two subtests of the production scale: one assessing sentence production, and one assessing expressive vocabulary. In the sentence subtest, children are required to make sentences of a similar structure to models given by the experimenter, to describe certain pictures, or arrays of toys. In the vocabulary subtest, children name objects or finish the experimenter’s sentences describing pictures. In addition to both tests, parents were asked to complete a Dutch version of the “Speech and Language Assessment Scale” (Hadley and Rice, 1993), in which they rated their child’s development on a variety of language skills compared to “other children of the same age,” starting from 1 (“very poor”) to 7 (“very good”).

## Results

On the standardized language tests, all of these children achieved scores within or above the normal range. Overall, the children have high LQs for comprehension (*m* = 115.4, SD = 11.8), for sentence production (*m* = 113.9, SD = 14.7), and for word production (*m* = 118.9, SD = 11.2). Their parents rate their average language skills also as somewhat above those of their peers (*m* = 4.7, SD = 0.9). The scores are highly correlated (see Table 2).

**Table 2:** **Correlation coefficients relating the language quotients and parental questionnaires at 3 years**.

	Sentence production LQ	Word production LQ	SLAS average
Comprehension LQ	0.577**	0.515*	0.499*
Sentence production LQ	–	0.411	0.669***
Word production LQ	–	–	0.326

***p < 0.001 **p < 0.01 *p < 0.05.

**Figure 6:**
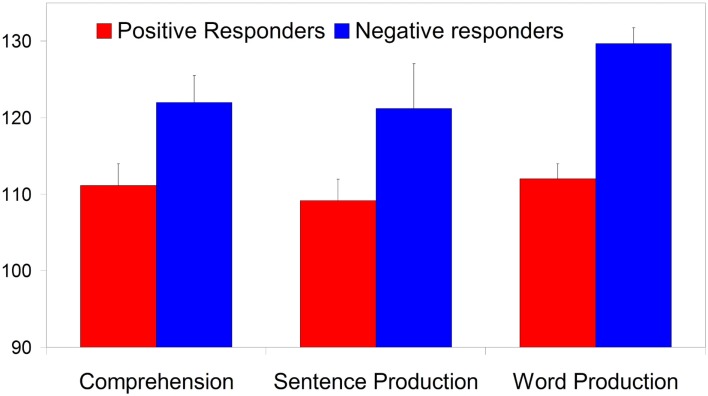
**The three language quotients at 3 years split by group performances at 7 months (error bars are one standard error from the mean)**. The group differences on comprehension and word production are significant (at *p* < 0.05 and *p* < 0.001 respectively), the sentence production difference just misses significance (*p* = 0.052).

Figure 6 shows that the Negative responders, with ERPs at 7 months resembling those of 10-month-olds, have significantly higher LQs than the Positive responders, whose ERPs at 7 months conformed to the overall seven-month group average. The Negative responders’ scores fall on average at 1.5 SD above the LQ mean, and the inter-group difference is significant for both comprehension (*t*_21_ = 2.37, *p* = 0.027) and word production (*t*_21_ = 5.85, *p* < 0.001), and almost significant for sentence production (*t*_21_ = 2.06, *p* = 0.052).

**Figure 7:**
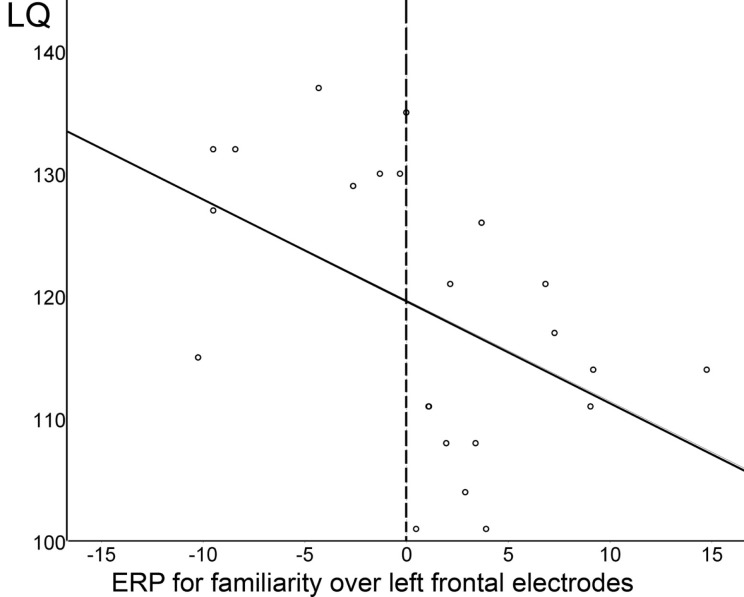
**The more negative the familiarity effect at 7 months (i.e. the more negative the difference wave between familiarized target and unfamiliar control words in the 350–450 ms time window over left frontal electrodes), the higher the quotient for word production at 3 years**. The dotted line indicates the split between Negative and Positive responders.

Further, across all 23 subjects, the ERP effect indexing speech segmentation ability at 7 months (i.e., difference between familiarized test and unfamiliar control words over left frontal electrodes in the 350–450 ms time window) and the LQ for word production at 3 years were significantly correlated, as can be seen in Figure 7: the more negative the difference wave, the higher the LQ for word production at 3 years (*r*_bivariate_ = −0.47, *p* = 0.02; with LQs for comprehension and sentence production partialed out, *r*_partial_ = −0.42, *p* = 0.06).

**Figure 8:**
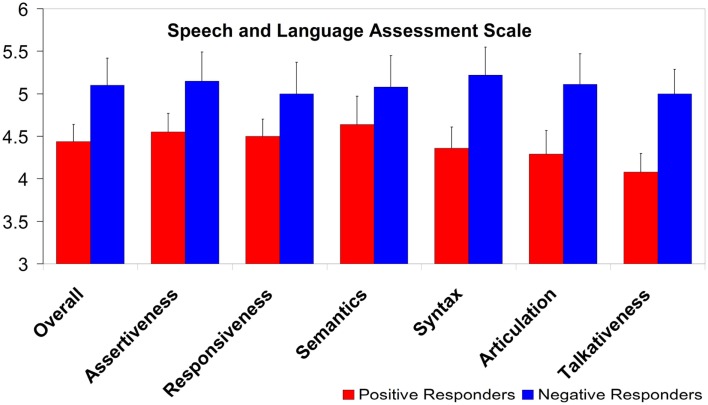
**Group ratings on the Speech and Language Assessment Scale, overall and per subscale**. A score of “4” corresponds to parents rating their child’s language performance as equal to their child’s peers; higher scores reflect better language ratings. (Error bars are one standard error from the mean).

Parents of Negative responders rated their children higher than parents of Positive responders did for their children (*t*_21_ = 1.86, *p* = 0.077). The average SLAS ratings, and separate group averages for each SLAS subscale, are shown in Figure 8; it can be seen that the Negative responders receive higher ratings in every case. The groups differ significantly on the syntax and talkativeness subscales (*t*_21_ = 2.09, *p* < 0.05, and *t*_21_ = 2.58, *p* < 0.02, respectively), and there is further a near-significant difference on the articulation subscale (*t*_21_ = 1.82, *p* = 0.084).

Together, these results show that ERPs for word recognition in continuous speech at 7 months are an indication of later language development. At 7 months the Negative responders delivered the brain response seen as a marker of segmentation in 10-month-olds. It is specifically in language processing that their brain responses differ from those of their age mates, and it is this specifically linguistic response that predicts their later vocabulary and sentence processing skills. Negative responders have higher language scores at 3 years than Positive responders, with the most marked difference being found for expressive vocabulary.

## Discussion

A 7-month-old’s brain responses in a segmentation task provide advance evidence of the later course of language proficiency development. At 3 years, infants who at 7 months had shown a left-lateralized negative-going brain response to a familiarized word in a sentence context linguistically outperformed infants who had shown a distributed positive-going brain response to the same stimuli at 7 months. The infant language skill difference appeared across a wide range of measures collected at 3 years, involving language at both the word and the sentence level, and skills in both speech comprehension and speech production.

Recall that our comparisons across the infant sub-groups had found that isolated-token repetition effects, as evidenced by change in response to the last two in comparison to the first two tokens in familiarization, did not differ for the Negative versus the Positive responders. Repetition effects are evidence of memory abilities (Rugg, 1985), and thus it would appear that the difference between our sub-groups is not one of simple memory capacity, but one with a more sharply linguistic focus: the test phase requires segmentation of the familiarized word from surrounding speech, and it is in this skill in particular that the Negative responder group outstrips their Positive responder age mates.

The significant differences that motivated a split into two sub-groups concerned only the brain response that signaled segmentation: the response time-locked to onset of the word that had been familiarized, when it was heard embedded in a sentence context. This response differed across the two sub-groups in both polarity and distribution, and the two sub-groups that were identified in this manner turned out to have significant differences in linguistic performance nearly 2.5 years later. Recall that Junge et al. (2012) also observed that it was individual differences in word recognition when words were presented in continuous speech, but not when presented in isolation, that were linked to future vocabulary. Together, these results strongly suggest that infant ERP responses of word recognition evidencing speech segmentation skill are predictive signals of linguistic development.

Mastery of segmentation is a fundamental skill indeed, because, as laid out in the introduction, most of the speech input an infant receives is in the form of multi-word utterances (Van de Weijer, 1999), and without being able to recognize words in these circumstances, the development of a substantial vocabulary cannot succeed. Segmenting speech into separate words forms part of the overall task of acquiring the phonology of the native language, in that the speech cues that inform lexical segmentation differ across languages. Evidence of segmentation in infant listening then appears earlier in some languages than in others, putatively for reasons of phonological salience and consistency of such segmentation cues. Mastering segmentation therefore rests on the construction of mental representations of language-specific phonology, prior to the availability of an extensive vocabulary from which such representations could have been abstracted.

It is perhaps little wonder that such a complex skill should vary in its rate of achievement across individuals. Such variation, and importantly, its relation to linguistic performance levels at 2 years, had already been demonstrated on the basis of behavioral measures both at a group level by Newman et al. (2006) and at an individual level by Singh et al. (2012). Moreover, related phonological skills of attunement to the native repertoire of phonetic contrasts have also been shown to vary across individuals and to be correlated with variation in later language skills (Kuhl et al., 2008); for instance, Tsao et al. (2004) measured the accuracy of vowel discrimination at 6 months, and also the speed with which a discrimination criterion could be reached, and found both measures to be predictive of vocabulary size in the second year of life. Although our results do not allow us to examine why it is that some infants displayed more mature speech segmentation skill than others, these findings further corroborate the proposition that such speech perception skills for the native language in infancy scaffold a child’s future language development (Cristia et al., submitted).

In the present study we have shown that the dimensions of inter-individual variation in early segmentation performance can be captured in terms of patterns of ERPs in infants’ brains. First, we have demonstrated that ERP evidence for segmentation is available earlier than behavioral evidence for the same skill. Although HPP studies with 7-month-olds acquiring Dutch had shown no significant evidence that segmentation of continuous speech was in place at that age (Kuijpers et al., 1998), ERP measurement detected such evidence where behavioral techniques could not. The onset of a familiarized word in a continuously spoken sentence context produced a brain response that had a significantly more positive amplitude than the response induced in matched contexts by a matched word that had not been previously presented in familiarization.

Interestingly, the overall pattern observed in these 7-month-old brains was not the same as had been observed in the measurements made somewhat later in the first year of life by Kooijman et al. (2005, 2009), Goyet et al. (2010), and Junge et al. (2012). In all those studies, brain responses to the familiar words were on average more negative than the responses to the matched unfamiliar control words. Kooijman et al. (2005) report this pattern both for the familiarization phase (with responses to the last tokens in the 10-token list being more negative than responses to the first tokens) and for the test phase (where the same difference contrasted the familiarized word against its matched control in the test set). The result in the familiarization condition of the present study with 7-month-olds also showed the same negative-going effect. But in the test condition of the present study, the overall average difference brain response was opposite in direction, with familiarity being associated with a more positive brain signal than unfamiliarity.

A positive familiarity effect for words in a continuous speech situation has in fact been reported before, in infants younger than our sample of 7-month-olds (6-month-olds, Männel and Friederici, 2010). This may suggest that ERP effects of word recognition in infancy gradually change from a positive (up to 6 months) to a negative polarity (from 10 months onward). However, we reiterate that brain maturation alone cannot explain the variation in polarity in the present sample across conditions. As we saw, at a group level the same 7-month-olds show a familiarity effect with negative amplitude for the isolated word familiarization phase. It is only in the test phase (requiring segmentation skill) that a positive familiarity effect is seen. Moreover, other studies have reported differential ERP responses across conditions within the same set of children, an asymmetry that brain maturation alone obviously cannot explain. For instance, Conboy and Mills (2006) showed that the relative dominance of a language in bilingual children explained the distribution of language-relevant ERP components. Junge et al. (2012) showed that the distribution of the word familiarity effect also hinges on the relative difficulty of the task, with a more focal distribution for the easier task (words introduced in isolation) and a broader distribution for the harder task (when words were introduced within an utterance).

Both polarity and distribution of the ERP effects played a role in distinguishing the two sub-groups of these 7-month-olds in the current study, with language skills at 3 years differing along with these earlier ERPs. The present study indicates that this variation is an important indicator of how the individual brains are performing the present linguistic processing task. In our 7-month-old participant group, a minority produced the negative-going effect (consistently seen across familiarization and test phases in the earlier studies with 10- to 12-month-olds) in the test phase as well as in familiarization. When assessed at 3 years of age, this minority then proved to deliver better sentence production and sentence comprehension performance, to have larger expressive vocabularies, and to receive higher ratings of their language skills from their parents, than the remaining majority group from the same 7-month-old participant population. The question prompted by these results is then: why do some 7-month-olds show a positive amplitude and others a negative amplitude?

Although our data set is limited in sample size, and we cannot do any source localization to derive any explanation about the origin of this polarity differences, a possible answer to the polarity issue can be found in other studies describing a similar phenomenon within the same age group. As described in the introduction, this is not the first occasion on which the same kind of significant ERP effect has been reported as negative under some conditions and as positive under others, even within the same age group. Both tone processing (Kudo et al., 2011) and phonetic discrimination (Garcia-Sierra et al., 2011) have been associated with such variation, and it has previously appeared in a segmentation-related task too (Männel and Friederici, 2010). Note further that ERP studies of early phonetic processing also used variation in polarity and distribution of responses to distinguish sub-groups within participant populations. Rivera-Gaxiola et al. (2005a) showed that differences in the patterns of 11-month-olds’ responses to non-native versus native contrasts were related to later word production abilities. In an oddball task with a constant standard, all children produced much the same negativity in response to a deviant differing across a native phoneme boundary (“native deviant”). Two sub-groups differed, however, in their response to another deviant that differed from the standard to the same degree as the native deviant but across a non-native phoneme boundary (“non-native deviant”). One sub-group produced a negativity in response to the non-native deviant too, with a parietal localization. The other sub-group produced a right fronto-central positivity, instead (thus effectively distinguishing the non-native and native contrasts in kind; Rivera-Gaxiola et al. (2005b) had also observed such sub-groups forming when they tracked the gradual attunement to native contrasts across the second half of the first year). The latter sub-group then proved to have developed larger productive vocabularies by 18 and continuing to 30 months of age.

Rivera-Gaxiola et al. (2005b) hypothesized that the polarity differences denote differences in auditory processing, with a positivity reflecting acoustic processing and a negativity reflecting more mature processing, possibly due to increased experience with the native language (Kuhl et al., 2008). This would entail for our study that the Positive responders relied on acoustic salience (of, for instance, the stressed syllable), whereas the Negative responders achieved word recognition with a more mature mechanism (i.e., segmenting fluent speech into word-like units). It is in this light noteworthy that a similar left-going negative marker of word recognition later in infancy has also been observed in studies comparing familiar/known versus unfamiliar/unknown isolated word processing (Mills et al., 1993, 1997, 2004, 2005; Thierry et al., 2003). As Junge et al. (2012) hypothesized, it is likely that for young infants, with a very small vocabulary, this same recognition mechanism indexing word meaning has developed from one that at a younger age is mainly sensitive to word form repetitions.

Our results indicate that a familiarity effect in infancy with negative amplitude in a speech segmentation task is associated with a more mature response, which in turn is associated with better language development. It would be interesting to examine whether infants who exhibit different polarities indexing word recognition in different circumstances (in isolation versus in multi-word utterances) also differ in the neural generators they use for word recognition, or in the way they use the same generators to achieve this. The use of neural networks could in turn also be affected by individual differences in brain maturation, in closing of the fontanels or by listening strategies. However, more research is clearly necessary to uncover the origin of individual variation in polarity and in distributions; our sample size is too small to draw final conclusions. Future research should also address the development of the word familiarity effect, not only within infancy, but also from infancy to adulthood, since a broad positive effect is again seen in many adult studies (Rugg, 1985; Snijders et al., 2007; but see Cunillera et al., 2006).

Finally, an additional contribution of the present study is clear evidence that the inter-group differences are longer-lasting than previously known. We retested our participants at age three and found wide-ranging evidence of language skills advantages for the group that had shown the 10-month-like ERP effect at 7 months. Thus we have reconfirmed the relation of early segmentation ability to later linguistic proficiency, and have shown that it lasts at least into the fourth year of life. Most importantly, though, we have isolated an ERP marker associated with differences in early segmentation ability. Infants who at 7 months already show an advanced marker of segmentation skill continue to develop better language skill at least through their third birthday.

## Conflict of Interest Statement

The authors declare that the research was conducted in the absence of any commercial or financial relationships that could be construed as a potential conflict of interest.

## Footnotes

^1^An ERP study uses more stimuli than an HPP study, and they are arranged differently (in ERP as in Table 1; in HPP, typically familiarization with two words, test with 6-sentence texts in which all sentences contain an instance of one of these words). Thus there were some differences between the present materials and those of Kuijpers et al.’s (1998) HPP study. When the present materials were adapted and tested in an HPP study with 7-month-olds, however, a null result was again observed (see Kooijman et al., 2008, for further detail).
